# The methyltransferase METTL3 promotes tumorigenesis via mediating HHLA2 mRNA m6A modification in human renal cell carcinoma

**DOI:** 10.1186/s12967-022-03496-3

**Published:** 2022-07-06

**Authors:** Dawei Zhu, Yingting Liu, Junjun Chen, Qi Wang, Yuan Li, Yulan Zhu, Jun Feng, Jingting Jiang

**Affiliations:** 1 Department of Tumor Biological Treatment, The Third Affiliated Hospital of Soochow University, Changzhou, 213003 Jiangsu People’s Republic of China; 2 Jiangsu Engineering Research Center for Tumor Immunotherapy, Changzhou, 213003 Jiangsu People’s Republic of China; 3 Institute of Cell Therapy, The Third Affiliated Hospital of Soochow University, Changzhou, 213003 Jiangsu People’s Republic of China; 4 State Key Laboratory of Pharmaceutical Biotechnology, Nanjing University, Nanjing, 210023 Jiangsu People’s Republic of China

**Keywords:** METTL3, HHLA2, m^6^A, ccRCC, Prognosis

## Abstract

**Background:**

As an important N6-methyladenosine (m^6^A) regulator, abnormal expression of methyltransferase-like protein 3 (METTL3) has been reported in certain human cancers. Although some data have shown that METTL3 plays an essential role in the progression of clear-cell renal cell carcinoma RCC (ccRCC), the detailed mechanism still remains largely undetermined.

**Methods:**

Immunohistochemistry (IHC) assay was used to examine the expression of METTL3 and its clinical implications in human ccRCC by using tissue-microarray (TMA). The cellular models based on ccRCC cell lines such as 786-O and ACHN, were established by operating METTL3 and HHLA2 via knockdown or overexpression, followed by in vitro cellular function studies and in vivo subcutaneous transplantation tumor model.

**Results:**

We found that METTL3 expression in ccRCC tissues was significantly higher compared with adjacent normal tissues. We also found the overall survival (OS) of the patients with low METTL3 expression was significantly better compared with the patients with high METTL3 expression. Furthermore, HHLA2^high^METTL3^high^ could serve as a better prognostic predictor for ccRCC patients. Depletion of METTL3 could significantly inhibit the cell viability, migration, and invasion abilities in ccRCC cell lines. Cellular studies further revealed that METTL3 could regulate HHLA2 expression via m^6^A modification of HHLA2 mRNA. In vitro studies revealed that HHLA2 overexpression could reverse the inhibition of cellular functions mediated by METTL3 depletion. The subcutaneous transplantation tumor model confirmed that HHLA2 overexpression could reverse the inhibition of tumor growth mediated by METTL3 depletion.

**Conclusion:**

Our study indicated that METTL3 served as an important prognostic predictor for ccRCC patients, and we demonstrated a novel regulatory mechanism of HHLA2 by mRNA epigenetic modification via METTL3. Moreover, we found that the METTL3/HHLA2 axis could promote tumorigenesis of ccRCC. Collectively, our current findings provided new insights into the therapeutic strategy against this malignancy targeting METTL3.

**Supplementary Information:**

The online version contains supplementary material available at 10.1186/s12967-022-03496-3.

## Background

The clear-cell renal cell carcinoma (ccRCC) accounts for more than 70% of all RCC cases worldwide [1]. Although routine surgical treatment remains the standard and recommended strategy for ccRCC, due to the highly immunogenic feature, the immunotherapeutic agents or even the novel combination strategies, have remarkably improved the overall survival (OS) of ccRCC patients in the recent years [2–5]. We have previously reported that as a novel therapeutic target, HHLA2 is overexpressed in human ccRCC tissues, and its expression is significantly associated with the prognosis and cancer progression of the patients [6]. However, the underlying regulatory mechanism of HHLA2 expression in human ccRCC remains largely undetermined.

Of note, like other important B7 family ligands, HHLA2 has been found to be overexpressed in lots of human cancers, and it can be used as a useful biomarker for the prediction of cancer progression and postoperative prognosis of the patients [7–10]. Recently, accumulative evidence has demonstrated that N6-methyladenosine (m^6^A), an important mechanism of post-transcriptional regulation, is involved in the regulation of B7 family ligands, such as PD-L1 and B7-H3 [11–13]. Wang et al. have demonstrated that most of the m^6^A RNA methylation regulators could be found abnormally expressed in ccRCC tissues, playing important roles in the initiation and progression of ccRCC, especially two powerful independent prognostic m^6^A RNA methylation regulators, METTL14 and METTL3 [14].

m^6^A is known as the most abundant mRNA modification catalyzed by a methyltransferase complex (MTC) [15]. The enzyme catalyzing the formation of m^6^A is called the m^6^A "writer" protein, and it has been described as a multi-component m^6^A MTC consisting of a heterodimer core of METTL3-METTL14 and other binding partners [16, 17]. The MTC core component of METTL3-METTL14 heterodimer catalyzes most m^6^A methylation in mRNA, and METTL3 is the only catalytic subunit with S-adenosine methionine (SAM) as the methyl donor [17]. Full-length METTL3 contains 580 amino acids, and it is composed of a zinc finger domain and a methyltransferase domain [18]. Studies have shown that METTL14 only plays a structural role in RNA binding and stabilization of the complex, while METTL3 is a catalytically active subunit with a cofactor binding pocket of SAM or S-adenosine homocysteine (SAH) [19]. The conserved DPPW motif (residue 395–399) of this enzyme is located in gate loop 1. When binding to SAM/SAH, gate loop 1 and gate loop 2 undergo a significant conformational change, resulting in cofactor binding pocket closure [19]. The levels of m^6^A variants have a variety of biological functions in mammals, including embryonic stem cell maintenance and differentiation, transcriptional splicing, nuclear RNA output, protein translation control, cell fate determination, and so on. And many studies have confirmed that m^6^A modification is closely associated with mRNA stability [20, 21].

Recently, increasing evidence has suggested that METTL3 plays a key role in many human cancers, either dependent or independent of its m^6^A RNA methyltransferase activity [15, 22–24]. Although the role of m^6^A modification in cancer has been extensively reviewed elsewhere, the key functions of METTL3 in various types of cancer and METTL3 as a potential target for cancer therapy have not been highlighted. Herein, we assessed the correlation between METTL3 expression and clinicopathological characteristics in ccRCC. Moreover, we explored the underlying mechanisms of METTL3 in the tumorigenesis of ccRCC. We found that HHLA2 acted as a critical target of METTL3, which might contribute to the oncogenic role of METTL3 in ccRCC.

## Materials and methods

### Tissue samples and patients

The ccRCC tissue array (Catalog No. HKidE180Su03) used in our present study was purchased from Shanghai Outdo Biotech Co., Ltd. (Shanghai, P. R. China). This ccRCC tissue array consisted of 90 patients (aged from 29 to 82 years, 59 males and 31 females). All these patients underwent surgery between October 2006 and February 2008. Several missing tissue points and incomplete tissue samples were excluded during the heat-induced antigen retrieval. Finally, a total of 84 cases were involved in the present study to evaluate the staining intensity of METTL3. Table 1 lists the detailed clinical parameters. All the tumor tissues were confirmed as ccRCC by hematoxylin and eosin (H&E) staining and pathological examination. Moreover, the staining results of HHLA2 have been reported in our previous study [6], and these results were also adopted to examine the association between METTL3 and HHLA2. The present study was approved by the ethics committee of our hospital.

**Table 1:** Correlation between the METTL3 expression and patients’ clinical parameters

Clinical parameters	Cases	METTL3 expression level		χ^2^	*P*
		H-score < 170	H-score ≥ 170		
Gender				0.382	0.536
Male	54	29	25		
Female	30	14	16		
Age (years)				1.810	0.179
≤ 60	47	21	26		
> 60	37	22	15		
Pathological stage				0.147	0.701
I + II	57	30	27		
III + IV	27	13	14		
Tumor size (diameter: cm)				3.827	0.051
< 5	40	16	24		
≥ 5	44	27	17		
Tumor position				0.186	0.666
Left	43	23	20		
Right	41	20	21		
T stage				0.026	0.871
I	54	28	26		
II + III	30	15	15		
Distance metastasis				2.149	0.143
M_0_	82	43	39		
M_1_	2	0	2		
TNM stage				0.825	0.364
I + II	78	41	37		
III + IV	6	2	4		

### Immunohistochemistry (IHC) and the evaluation of METTL3 immunostaining

IHC was performed to characterize the expression of METTL3 in ccRCC tissues and adjacent normal tissues. In brief, paraffin-embedded tissue chips were dried at 90 °C for 4 h, dewaxed in xylene, and then rehydrated in a series of graded ethanol solutions. EDTA solution (1 mM, pH 9.0) was used for antigen retrieval. 0.3% hydrogen peroxide solution was used to block endogenous peroxidase activity for the tissue sections which followed by rinsing with PBS for 5 min, and blocking with 3% BSA solution. The sections were then incubated with the primary antibody (rabbit anti-human METTL3 monoclonal antibody, catalog No. ab195352, Abcam, used in 1:150) at 4 °C overnight, followed by incubation with HRP-labeled secondary antibody at 37 °C for 30 min. Diaminobenzene was used as the chromogen, and hematoxylin was used as the nuclear counterstain. Sections were then dehydrated, cleared, and mounted. The immunostaining intensity of METTL3 was assessed according to the *H-score* method as previously described [25, 26]. The *H-scores* ranged from 0 (100% negative tumor cells) to 300 (100% strongly stained tumor cells). The results were then recorded and used for further statistical analysis.

### Cell lines, and cellular studies of proliferation, invasion and migration

The human ccRCC cell lines ACHN and 786-O, were purchased from the Chinese Academy of Sciences, Shanghai Institutes for Biological Sciences (Shanghai, China). The cells were maintained in DMEM supplemented with 10% fetal bovine serum (FBS) in the presence of benzylpenicillin (100 U/mL), streptomycin (100 μg/mL), and 2 mM L-glutamine under standard culture conditions (5% CO_2_, 37 °C). The cellular studies including proliferation and invasion were carried out according to our previous study [6]. The wound healing assay used to evaluate the migration ability of different cell lines was performed, and the results were analyzed according to the ImageJ method from the published reports [27, 28].

### Establishment of stable METTL3 knockdown and HHLA2 overexpression renal cancer cell lines

To construct stable METTL3 knockdown renal cancer cell lines, shRNA (5ʹ-CAGGAGATCCTAGAGCTATTAAATATTCAAGAGATATTTAATAGCTCTAGGATCTCCTGTTTTTTG-3ʹ) specific targeting to *METTL3* was designed by Invitrogen online tool (https://rnaidesigner.thermofisher.com/rnaiexpress/) and cloned into the lentiviral pLVX-sh1 vector. Lentivirus carrying METTL3 shRNA was constructed by Genelily Biotech Co.,LTD (Shanghai, China). LV-NC represents an empty vector packaged by lenti-virus as negative controls. The 786-O and ACHN cell lines in the 6-well plate were infected with the lentivirus following the manufacturer’s instruction. After 24 h, the medium was replaced with a complete medium. The stably infected renal cancer cells were selected by incubation with 2 μg/ml of puromycin for two weeks. The efficiency of METTL3 knockdown was examined by qRT-PCR and Western blotting. To construct stable HHLA2 overexpression renal cancer cell lines with or without METTL3 knock-down, the human HHLA2 coding sequences (NM_007072.3) were purchased from Generalbiol Biotech (Hefei, Anhui, China) and cloned into the lentiviral pLVX-IRES-ZsGreen vector. Lentivirus carrying HHLA2 coding sequences were constructed by Genelily Biotech Co.,LTD (Shanghai, China). The vector control or METTL3 knock-down cell lines were seeded in a 6-well plate and infected with the lentivirus following the manufacturer’s instruction. After 24 h, the medium was replaced by using the complete medium. The stably infected ZsGreen renal cancer cells were isolated by fluorescence-activated cell sorting (FACS). The efficiency of HHLA2 overexpression was then examined by qRT-PCR and Western blotting.

### Real-time PCR

RNA extraction and Real-time PCR were performed as previously described [6]. Briefly, the relative mRNA levels of the indicated genes were calculated by the 2^−ΔΔCT^ method. *GAPDH* was used as a housekeeping gene. Primer sequences were as follows: *METTL3*, 5ʹ-CAAGCTGCACTTCAGACGAA-3ʹ(Forward); 5ʹ-GCTTGGCGTGTGGTCTTT-3ʹ(Reverse); *HHLA2*, 5ʹ-TGCCCTCTGCGATTTTGGCA-3ʹ(Forward); 5ʹ-GGCTCCATCAGCAGGGTGTC-3ʹ(Reverse); *GAPDH*, 5ʹ-TGACTTCAACAGCGACACCCA-3ʹ(Forward); 5ʹ-CACCCTGTTGCTGTAGCCAAA-3ʹ(Reverse).

### Western blotting analysis

The Western blotting analysis was performed as previously described [6]. The antibodies against METTL3 (1:2,000; Catalog No. ab195352, Abcam, MA, USA), HHLA2 (1:2,000; Catalog No. ab214327, Abcam, MA, USA), and GAPDH (1:4,000, Sigma, St. Louis, MO, USA) were used in the present study, and the HRP-labeled goat anti-mouse/rabbit secondary antibody (1:6,000) was purchased from Sigma Aldrich (St. Louis, MO, USA). The immunoreactive bands were examined by an enhanced chemiluminescence detection kit (Thermo Fisher, MA, USA), and then exposed to X-ray film, and band densities were quantified by densitometry with a video documentation system (Gel Doc 2000, Bio-Rad).

### RNA m^6^A methylation quantification

The m^6^A RNA Methylation Assay Kit (Abcam, ab185912) was used to evaluate the content of m^6^A in total RNA as previously reported [29]. Briefly, 300 ng RNA accompanied with m^6^A standard was coated on assay wells, followed by the addition of capture antibody solution and secondary detection antibody solution. The colorimetric m^6^A levels were quantified by reading the absorbance of each well at OD450, and then calculated based on the standard curve.

### Me-RIP assay

The methylated m^6^A RNA immunoprecipitation (Me-RIP) was performed as previously described. Briefly, total RNA was isolated from RCC cells by the Trizol method and added into RIPA buffer at 4 °C. Then the RNA samples were incubated with 5 μg anti-m^6^A antibody, or IgG (Abcam) pre-conjugated protein A/G Magnetic Beads (Merck Millipore) in 500 μL IP buffer supplemented with 100 units of RNase inhibitors (Thermo Fisher) at 4 °C overnight. The IP complex was treated with proteinase K (Thermo Fisher) at 52 °C for 1 h. Finally, the methylated HHLA2 RNA was evaluated by qRT-PCR.

### RNA stability assay

Actinomycin D (5 μg/ml, A9415, Sigma-Aldrich) was added to RCC cells to assess RNA stability. After incubation for indicated time points (0, 3, and 6 h), the cells were collected and RNA samples were extracted for qPCR.

### Subcutaneous transplantation model

Female Balb/c nude mice (4 ~ 5 weeks old) were bred under an aseptic-specified pathogen-free (SPF) condition. Animal experiments were approved by the Animal Care and Use Committee and complied with the Guidelines on Animal Welfare of the China National Committee for Animal Experiments. The 786-O cells (1 × 10^7^) from LV-NC, LV-METTL3-shRNA, LV-NC + LV-HHLA2^OE^, and LV-METTL3-shRNA + LV-HHLA2^OE^ suspended in 0.15 mL PBS were subcutaneously injected in the right inguinal region of nude mice separately. Five mice were used for each group. Subsequently, tumor sizes were determined every 5 days with a caliper, and the volume was calculated using the formula as follows: length × width^2^ × 0.5.

### Statistical analysis

Statistical analyses were performed using Prism 8 software (GraphPad). Chi-square test, Two-way ANOVA, paired or unpaired *t*-test, and Log-rank survival analysis, was used where needed. The in vitro data represented at least three independent experiments. *P* < 0.05 was considered statistically significant.

## Results

### Correlations between the expressions of METTL3 and HHLA2 in human ccRCC tissues

**Fig. 1:**
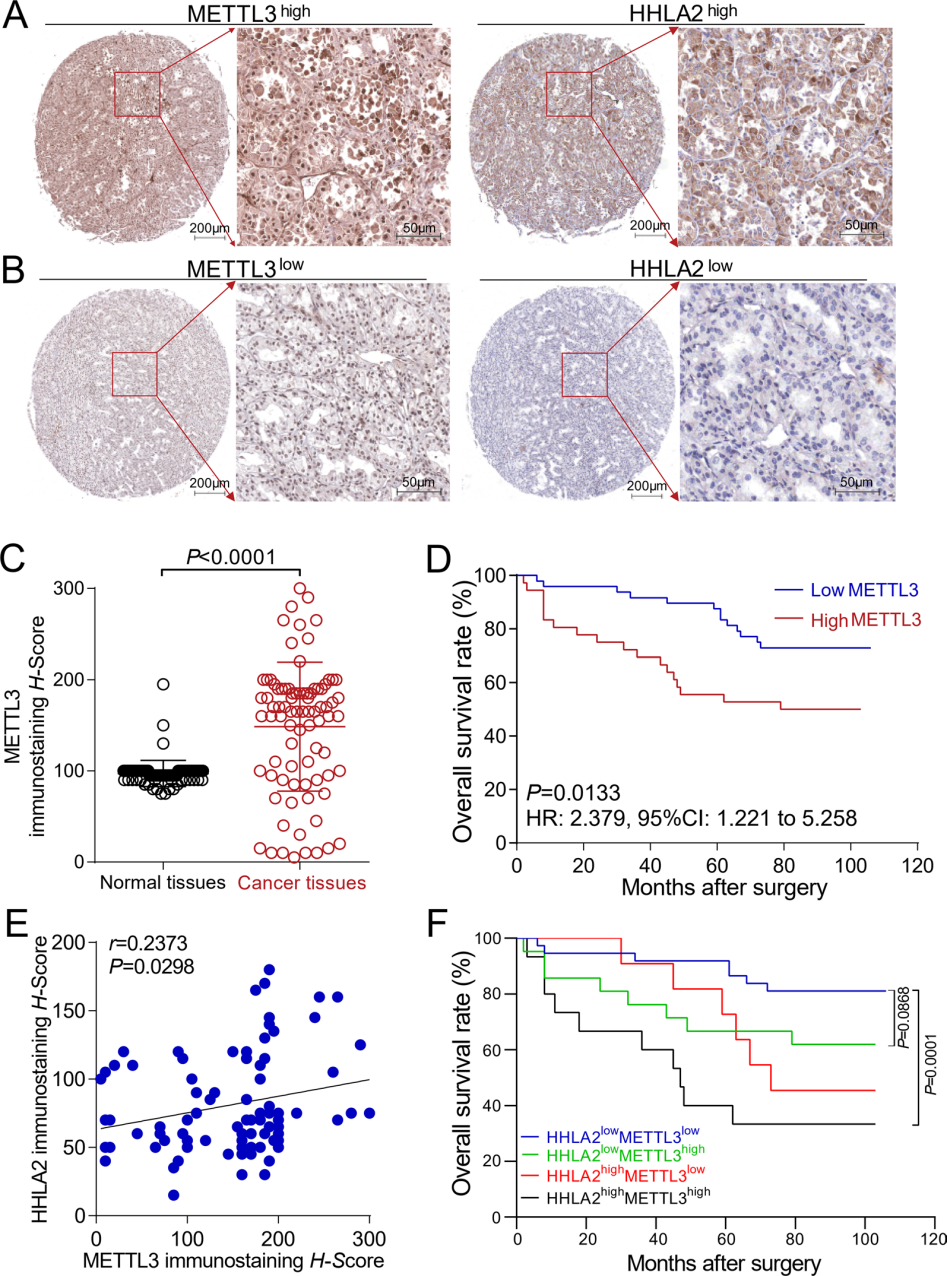
Immunostaining of METTL3 in human ccRCC tissues and its correlation with HHLA2 expression. **A** Higher METTL3 expression was found in human ccRCC tissue and predominantly localized in the nucleus of cancer cells, and in the serial section of ccRCC tissue, higher expression of HHLA2 was also found and predominantly localized in the cytoplasm of cancer cells (scale bar = 200 µm or 50 µm). **B** Lower expression of METTL3 was found in human ccRCC tissue, and in the serial section of ccRCC tissue, lower expression of HHLA2 was also found (scale bar = 200 µm or 50 µm). **C** The expression of METTL3 in ccRCC tissues was significantly higher compared with the adjacent normal tissues (*P* < 0.0001). **D** The OS of the patients with low expression of METTL3 was significantly better compared with the patients with its high expression (*P* = 0.0133, HR: 2.379, 95% CI 1.221 to 5.258). **E** The expression of METTL3 in ccRCC tissues was significantly correlated with the HHLA2 expression (*r* = 0.2373, *P* = 0.0298). **F** The OS of the HHLA2^low^METTL3^low^ patients was significantly better compared with the HHLA2^high^METTL3^high^ patients (*P* = 0.0001), and the OS of the HHLA2^low^METTL3^low^ patients trended better than the HHLA2^low^METTL3^high^ patients (*P* = 0.0868). Paired *t* test, Log-rank survival analysis and Spearman correlation analyses were used respectively

The human ccRCC tissue array and IHC assay were used to examine the expression of METTL3. The detailed data of HHLA2 expression in the serial section of this human ccRCC tissue-array block have been reported in our previous study [6], which were then used in the correlation analysis of METTL3 and HHLA2. Figure 1A and B show that METTL3 was predominantly localized in the nucleus of the cancer cells, while HHLA2 was predominantly localized in the cytoplasm. Figure 1C indicates that the expression of METTL3 in ccRCC tissues was significantly higher compared with adjacent normal tissues (*P* < 0.0001). The Additional file 1: Figure S1A and B also shows that moderate METTL3 expression and weak HHLA2 expression could be found in adjacent normal tissues. The OS of patients with low expression of METTL3 was significantly compared with the patients with its high expression (*P* = 0.0133, HR: 2.379, 95% CI 1.221 to 5.258, Fig. 1D). Figure 1E shows that the expression of METTL3 in ccRCC tissues was significantly correlated with the expression of HHLA2 (*r* = 0.2373, *P* = 0.0298). Moreover, based on the combined expression of METTL3 and HHLA2, the survival analysis reveals that the OS of the HHLA2^low^METTL3^low^ patients was significantly better compared with the HHLA2^high^METTL3^high^ patients (*P* = 0.0001, Fig. 1F), and the OS of the HHLA2^low^METTL3^low^ patients trended better than the HHLA2^low^METTL3^high^ patients (*P* = 0.0868, Fig. 1F). Although we did not find any significant association between the expression of METTL3 and the clinical parameters of the patients (Table 1), the COX model analysis results showed that the expression of METTL3 could be used as an important and independent prognostic risk factor for ccRCC patients (HR = 4.071, *P* = 0.003, 95% CI 1.624 ~ 10.208, Table 2).

**Table 2:** COX model analysis based on METTL3 expression and patients’ clinical parameters

Variables	HR (95% CI)	*P*	HR (95% CI)	*P*
Gender (male: female)	0.929 (0.451 ~ 1.914)	0.842	1.723 (0.763 ~ 3.888)	0.190
Age (> 60: ≤ 60 years)	3.117 (1.465 ~ 6.634)	**0.003**	5.652 (2.348 ~ 13.609)	**0.000**
Pathological stage (III + IV: I + II)	5.028 (2.423 ~ 10.435)	**0.000**	5.799 (2.389 ~ 14.075)	**0.000**
Tumor size (≥ 5: < 5 cm)	1.732 (0.829 ~ 3.618)	0.144	0.991 (0.373 ~ 2.639)	0.986
Tumor position (right: left)	0.950 (0.469 ~ 1.921)	0.885	0.809 (0.366 ~ 1.790)	0.601
T stage (II + III: I)	3.544 (1.731 ~ 7.259)	**0.001**	3.849 (1.530 ~ 9.688)	**0.004**
Distance metastasis (M_1_: M_0_)	8.882 (1.954 ~ 40.372)	**0.005**	3.745 (0.367 ~ 38.232)	0.265
TNM stage (III + IV: I + II)	3.433 (1.193 ~ 9.879)	**0.022**	1.239 (0.256 ~ 5.986)	0.790
METTL3 expression (high: low)	2.379 (1.221 ~ 5.258)	**0.013**	4.071 (1.624 ~ 10.208)	**0.003**

Bold signifies *P* < 0.05

### Establishment of stable depletion of METTL3 in ccRCC cell lines 786-O and ACHN

**Fig. 2:**
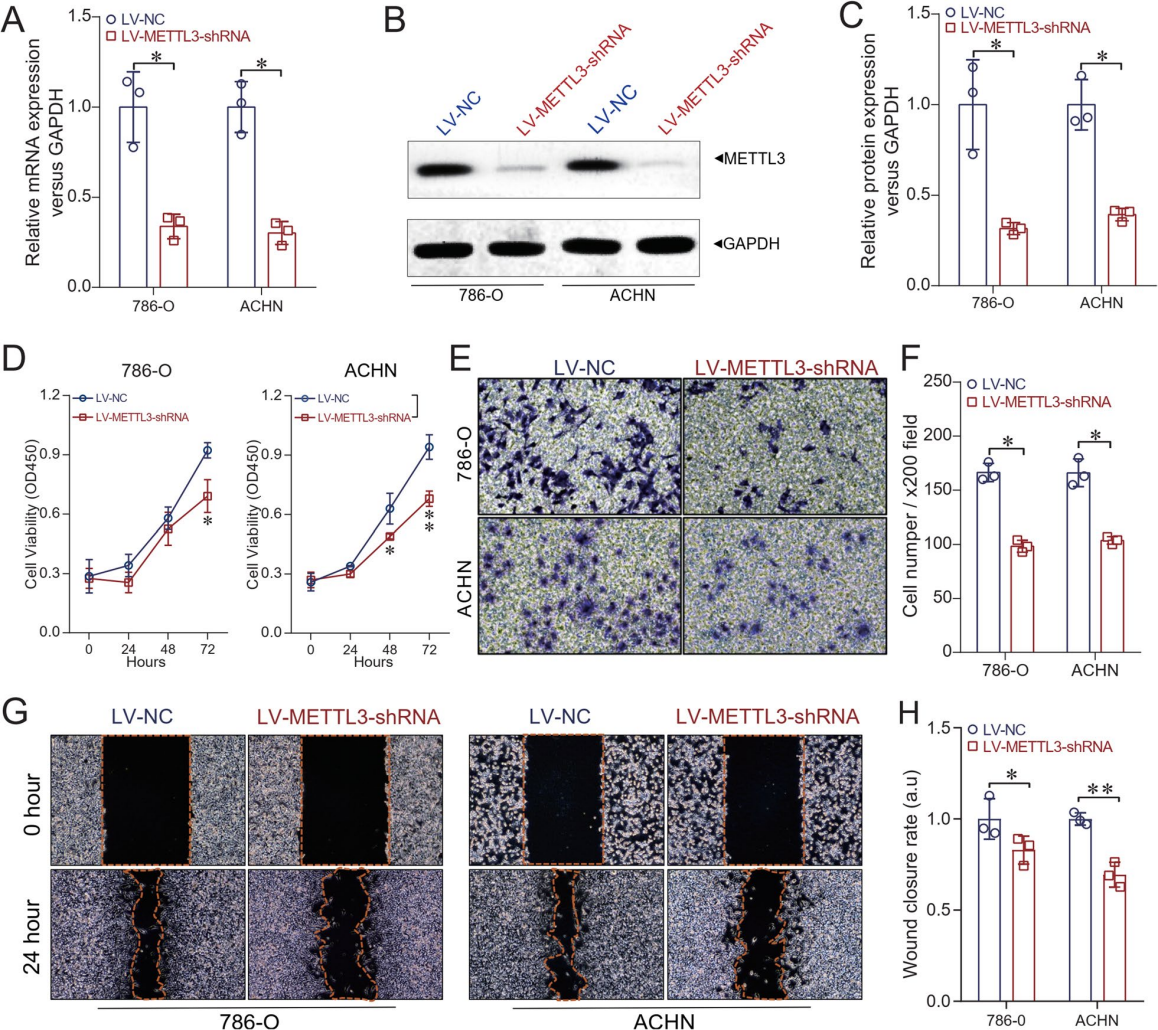
Depletion of METTL3 expression in ccRCC cell lines. The stable depletion model of METTL3 in ccRCC cell lines 786-O and ACHN was established by using the RNAi method. **A** qRT-PCR results showed that the expression of METTL3 at the mRNA level was significantly decreased in LV-METTL3-shRNA compared with LV-NC in both 786-O and ACHN cells (*P* < 0.05 respectively). **B** and **C** Western blot analysis showed that METTL3 expression protein level was significantly decreased in LV-METTL3-shRNA compared with LV-NC in both 786-O and ACHN cells (*P* < 0.05 respectively). **D** CCK-8 assay showed that the cell proliferation ability of LV-METTL3-shRNA was significantly inhibited compared with the LV-NC (*P* < 0.05 in 786-O cells at 72 h, *P* < 0.05 at 48 h and *P* < 0.01 at 72 h respectively in ACHN cells). **E** and **F** Transwell assay results revealed that the cell invasion ability of LV-METTL3-shRNA was significantly decreased compared with LV-NC (*P* < 0.05 in 786-O cells, and *P* < 0.05 in ACHN cells). **G** and **H** Wound-healing assay showed that at the time point of 24 h, the relative wound closure rate of LV-METTL3-shRNA was significantly decreased compared with LV-NC (*P* < 0.05 in 786-O and *P* < 0.01 in ACHN cells, respectively). Un-paired *t* test was used as needed. ^*^P < 0.05, ^**^P < 0.01

In our present study, lentivirus-mediated short hairpin RNA (shRNA) interference targeting METTL3 was used to establish the stable depletion of METTL3 in ccRCC cell lines 786-O and ACHN. Figure 2A shows that the expression of METTL3 at the mRNA level was significantly decreased in LV-METTL3-shRNA compared with LV-NC in both 786-O and ACHN cells (*P* < 0.05). Moreover, we also found that the expression of METTL3 at the protein level was significantly decreased in LV-METTL3-shRNA compared with LV-NC in both 786-O and ACHN cells (*P* < 0.05, Fig. 2B and C). The CCK-8 assay also showed that the proliferation ability of LV-METTL3-shRNA cells was significantly inhibited compared with LV-NC cells (*P* < 0.01 in 786-O, and *P* < 0.05 in ACHN, Fig. 2D). In addition, the transwell assay revealed that the invasion ability of LV-METTL3-shRNA cells was significantly decreased compared with LV-NC cells (*P* < 0.05 in 786-O, and *P* < 0.05 in ACHN, Fig. 2E and F). Besides, we also evaluated the migration ability of the ccRCC cell lines after depletion of METTL3 and found that at the time point of 24 h, the relative wound closure rate of LV-METTL3-shRNA (*P* < 0.05 in 786-O and *P* < 0.01 in ACHN cells, Fig. 2G and H) was significantly increased compared with LV-NC, suggesting that depletion of METTL3 significantly reduced the migration ability of ccRCC cell lines.

### METTL3 regulates the expression of HHLA2 via m^6^A modification of HHLA2 mRNA.

**Fig. 3:**
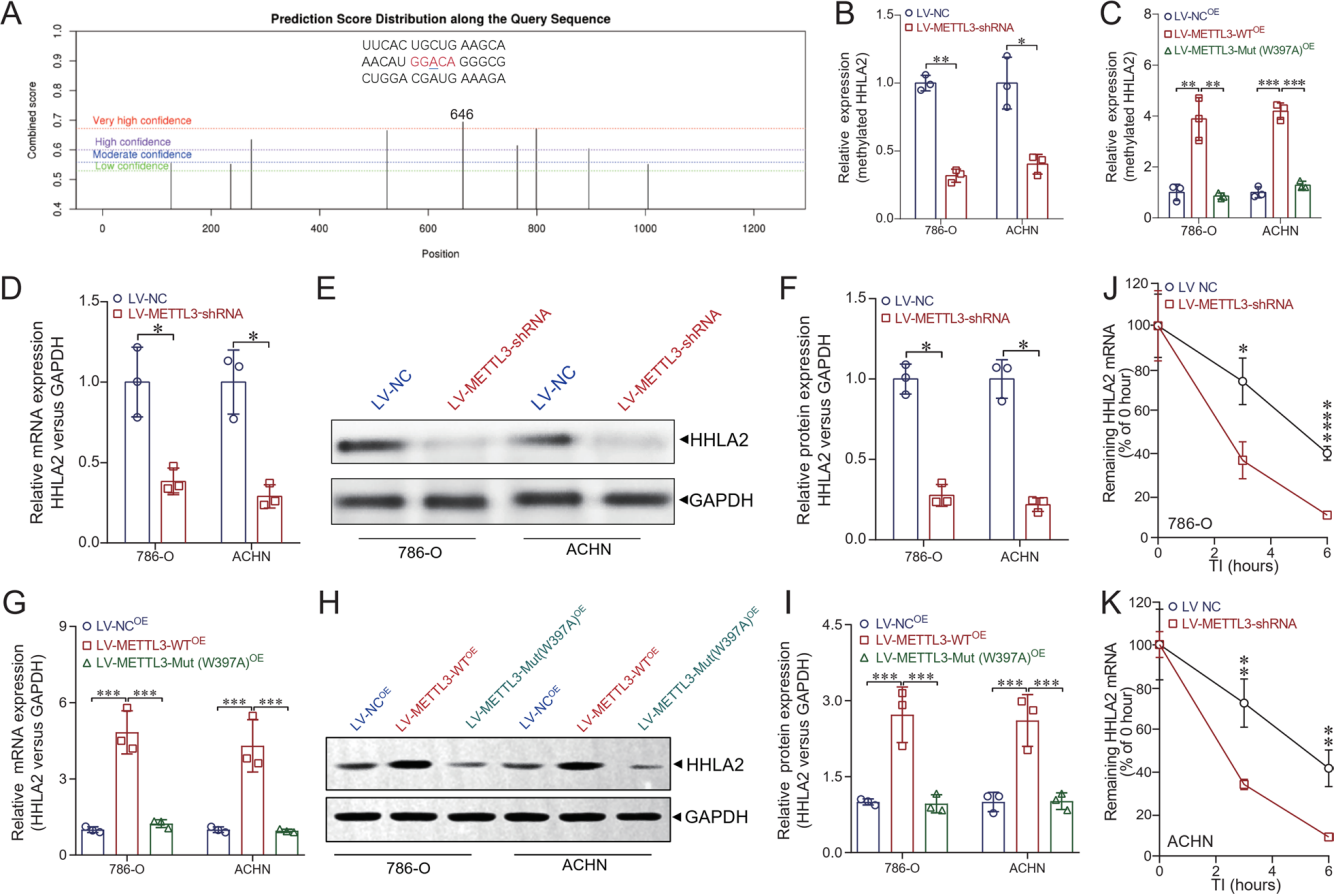
METTL3 regulates the expression of HHLA2 via m^6^A modification of HHLA2 mRNA. **A** The potential m^6^A site of HHLA2 mRNA was analyzed via a sequence-based m^6^A modification site predictor (SRAMP, http://www.cuilab.cn/sramp), and we found a very high confidence m^6^A site in HHLA2 mRNA (GGACA). **B** m^6^A-labeled mRNA level of HHLA2 was decreased in LV-METTL3-shRNA cells compared with LV-NC cells (*P* < 0.01 in 786-O and *P* < 0.05 in ACHN respectively). **C** m^6^A-labeled mRNA levels of HHLA2 were significantly elevated in METTL3-overexpressing ccRCC cells (*P* < 0.01 in 786-O and *P* < 0.001 in ACHN), but not in METTL3-Mut (W397A)-overexpressing ccRCC cells. **D** HHLA2 mRNA expression level was significantly decreased in LV-METTL3-shRNA compared with LV-NC in both 786-O and ACHN cells (*P* < 0.05). **E** and **F** Western blot analysis showed that HHLA2 protein expression level was significantly decreased in LV-METTL3-shRNA compared with LV-NC in both 786-O and ACHN cells (*P* < 0.05 respectively). **G** HHLA2 mRNA expression level was significantly increased in METTL3-overexpressing ccRCC cells (*P* < 0.001 in 786-O and ACHN respectively), but not in METTL3-Mut (W397A)-overexpressing ccRCC cells. **H** and **I** Western blot analysis showed that HHLA2 protein expression level was significantly increased in METTL3-overexpressing ccRCC cells (*P* < 0.001 in 786-O and ACHN respectively), but not in METTL3-Mut (W397A)-overexpressing ccRCC cells. **J** and **K** Changes of HHLA2 mRNA expression and decay rate after transcription inhibition were confirmed by qRT-PCR in the 786-O and ACHN cell lines when METTL3 was depleted. Un-paired *t* test was used as needed. ^*^P < 0.05, ^**^P < 0.01, ^***^P < 0.001, ^****^P < 0.0001

We have previously reported that HHLA2 was highly expressed in human ccRCC tissues, and potentially involved in the promotion of cancer progression [6]. Thus the underlying mechanism of the regulation of HHLA2 expression in ccRCC cell lines merits further investigation. Herein, we predicted the potential m^6^A site of HHLA2 mRNA via a sequence-based m^6^A modification site predictor (SRAMP, http://www.cuilab.cn/sramp) and found a very high confidence m^6^A site in HHLA2 mRNA (GGACA) (Fig. 3A). Figure 3B shows that the m^6^A-labeled mRNA of HHLA2 was significantly impaired in ccRCC cell lines (*P* < 0.01 in 786-O and *P* < 0.05 in ACHN). We also noticed that m^6^A-labeled mRNA levels of HHLA2 were significantly elevated in METTL3-overexpressing ccRCC cells (*P* < 0.01 in 786-O and *P* < 0.001 in ACHN, Fig. 3C). We also generated a mutated METTL3 (W397A) construct with disordered enzymatic activity as described previously [16] and found that mutant METTL3 failed to elevate the m^6^A methylation level of HHLA2 mRNA in ccRCC cells (*P* < 0.01 in 786-O and *P* < 0.001 in ACHN, Fig. 3C). As shown in Fig. 3D, we also found that the HHLA2 mRNA expression level was significantly decreased in LV-METTL3-shRNA compared with LV-NC in both 786-O and ACHN cells. Western blot analysis also showed that HHLA2 protein expression level was significantly decreased in LV-METTL3-shRNA compared with LV-NC in both 786-O and ACHN cells (Fig. 3E and F). Moreover, we also found HHLA2 mRNA (Fig. 3G) and protein (Fig. 3H and I) expression levels were significantly increased in METTL3-overexpressing ccRCC cells, but not in METTL3-Mut (W397A)-overexpressing ccRCC cells, suggesting the regulation of METTL3 on HHLA2 expression dependent on the m^6^A methylation ability of METTL3. Furthermore, decreased HHLA2 mRNA expression and mRNA stability were confirmed by qRT-PCR in the 786-O and ACHN cell lines when METTL3 was depleted (Fig. 3J and K). Collectively, these results suggested that METTL3 epigenetically elevated the expression of HHLA2 at the mRNA level in ccRCC.

### In vitro studies reveal that HHLA2 overexpression can reverse the inhibition of cellular functions mediated by METTL3 depletion

**Fig. 4:**
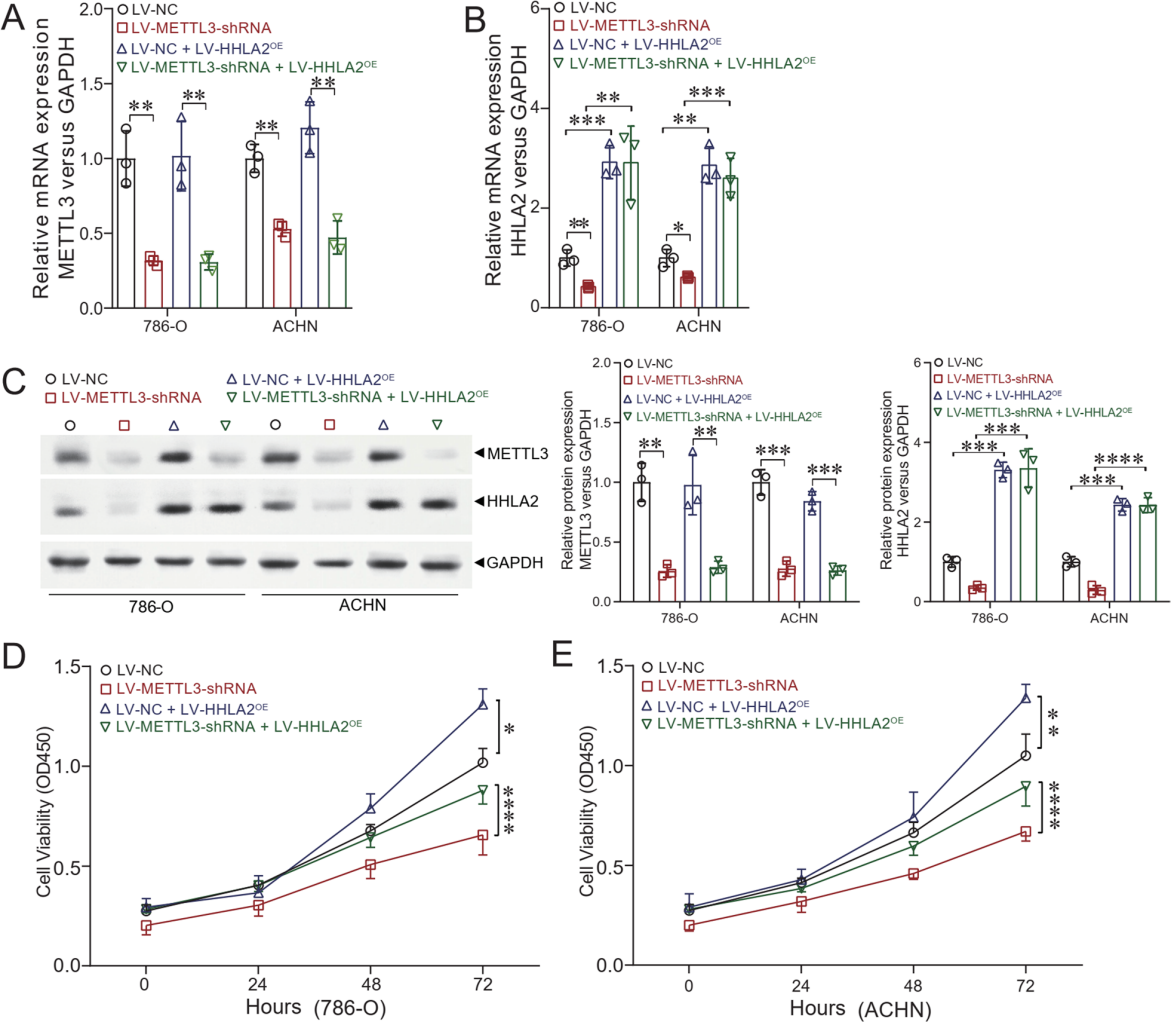
In vitro studies revealed that HHLA2 overexpression could reverse the inhibition of cellular functions mediated by METTL3 depletion. We further constructed the HHLA2 overexpression cellular models based on the METTL3 depletion in 786-O and ACHN cells. LV-NC, LV-METTL3-shRNA, LV-NC + LV-HHLA2^OE^, and LV-METTL3-shRNA + LV-HHLA2^OE^ cells were involved in the present study. **A** and **B** Based on the qRT-PCR results, the expression of METTL3 at the mRNA level was significantly decreased after METTL3 depletion, and HHLA2 mRNA expression level was significantly increased after HHLA2 overexpression upon METTL3 depletion. **C** Based on the Western blot analysis, METTL3 protein expression was significantly decreased after METTL3 depletion, and HHLA2 protein expression level was significantly increased after HHLA2 overexpression upon METTL3 depletion. **D** In 786-O cells, after depletion of METTL3, overexpression of HHLA2 could significantly increase the cellular viability. **E** In ACHN cells, after depletion of METTL3, overexpression of HHLA2 could significantly increase the cellular viability. Un-paired *t* test and Two-way ANOVA analyses were used respectively. ^*^P < 0.05, ^**^P < 0.01, ^***^P < 0.001, ^****^P < 0.0001

**Fig. 5:**
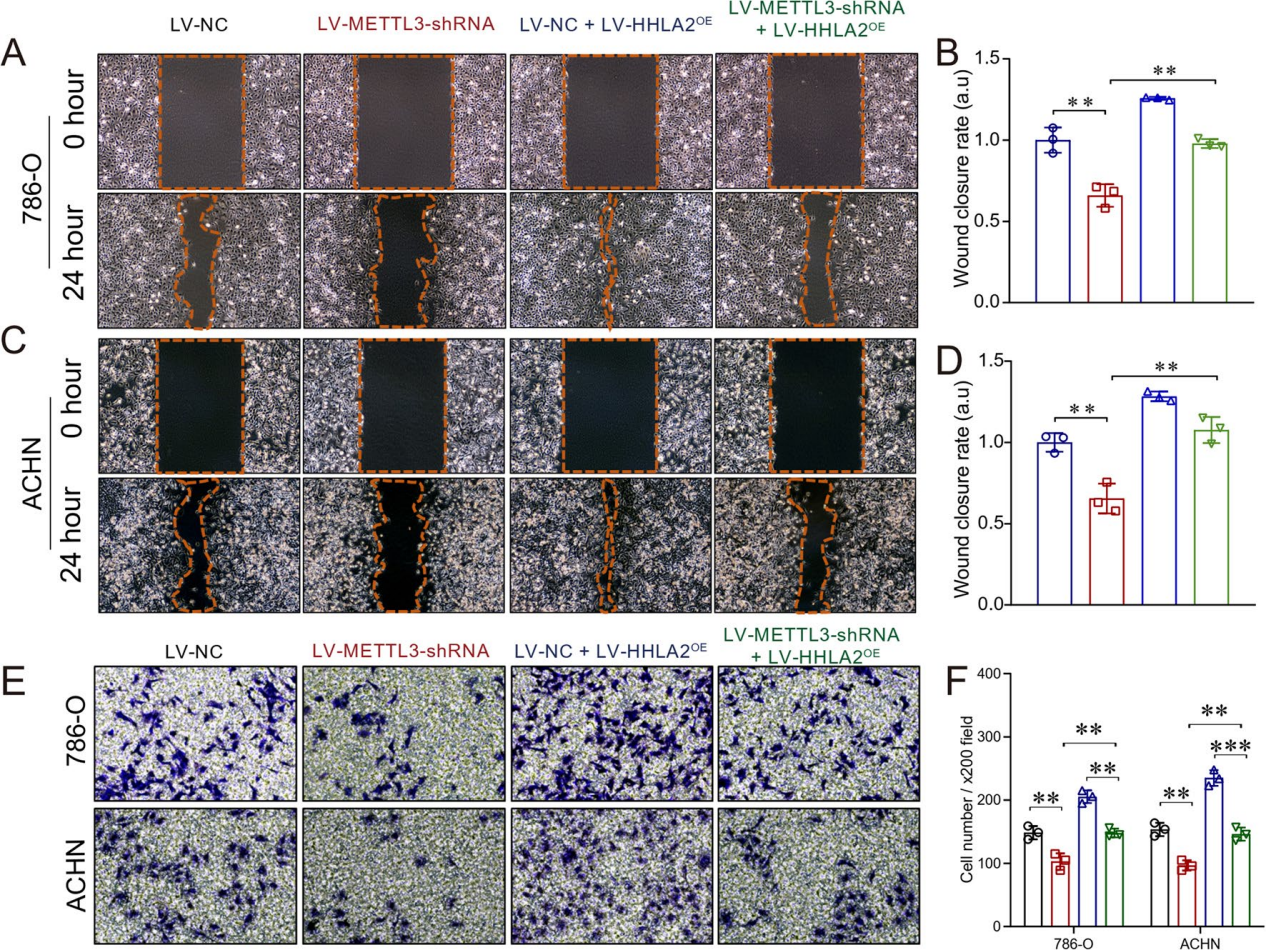
In vitro studies revealed that overexpression of HHLA could significantly increase the cellular migration abilities by using wound-healing assay and transwell assay. **A** and **B** The wound-healing assay revealed that in 786-O cells, after depletion of METTL3, overexpression of HHLA2 could significantly increase the cellular migration abilities (LV-METTL3-shRNA *v.s*. LV-METTL3-shRNA + LV-HHLA2^OE^, *P* < 0.01). **C** and **D** The wound-healing assay revealed that in ACHN cells, after depletion of METTL3, overexpression of HHLA2 could significantly increase the cellular migration abilities (LV-METTL3-shRNA *v.s*. LV-METTL3-shRNA + LV-HHLA2^OE^, *P* < 0.01). **E** and **F** The transwell assay results confirmed that, in both 786-O and ACHN cells, overexpression of HHLA2 could significantly increase the cellular invasion abilities (LV-METTL3-shRNA *v.s*. LV-METTL3-shRNA + LV-HHLA2^OE^, *P* < 0.01 respectively). Un-paired *t* test was used as needed. ^**^P < 0.01, ^***^P < 0.001

To further study whether HHLA2 overexpression could reverse the inhibition of cellular functions mediated by METTL3 depletion, we constructed the HHLA2 overexpression cellular models based on METTL3 depletion in 786-O and ACHN cells. Figure 4A and B show that the expressions of METTL3 and HHLA2 at the mRNA level were confirmed by qRT-PCR, verifying that we successfully established the cellular models. Moreover, Fig. 4C shows that the expressions of METTL3 and HHLA2 at the protein levels were confirmed by Western blot analysis, further verifying that we successfully established the cellular models. In addition, the CCK-8 assay (Fig. 4D and E) showed that overexpression of HHLA2 could significantly increase the cellular viability in both 786-O and ACHN cells (*P* < 0.05 and *P* < 0.01, respectively). And also overexpression of HHLA2 could significantly reverse the inhibition of cellular viability upon METTL3 knockdown in both 786-O and ACHN cells (*P* < 0.0001 respectively). The wound-healing assay also revealed that overexpression of HHLA2 could significantly increase the cellular migration abilities upon METTL3 knockdown in both 786-O and ACHN cells (LV-METTL3-shRNA *v.s*. LV-METTL3-shRNA + LV-HHLA2^OE^, *P* < 0.01 respectively, Fig. 5A, B, C and D). Furthermore, we also carried out the transwell assay, and the results confirmed that overexpression of HHLA2 could significantly increase the cellular invasion abilities upon METTL3 knockdown in both 786-O and ACHN cells (LV-METTL3-shRNA *v.s*. LV-METTL3-shRNA + LV-HHLA2^OE^, *P* < 0.01 respectively, Fig. 5E and F).

### Subcutaneous transplantation tumor model identities that HHLA2 overexpression can reverse the inhibition of tumor growth induced by depletion of METTL3

**Fig. 6:**
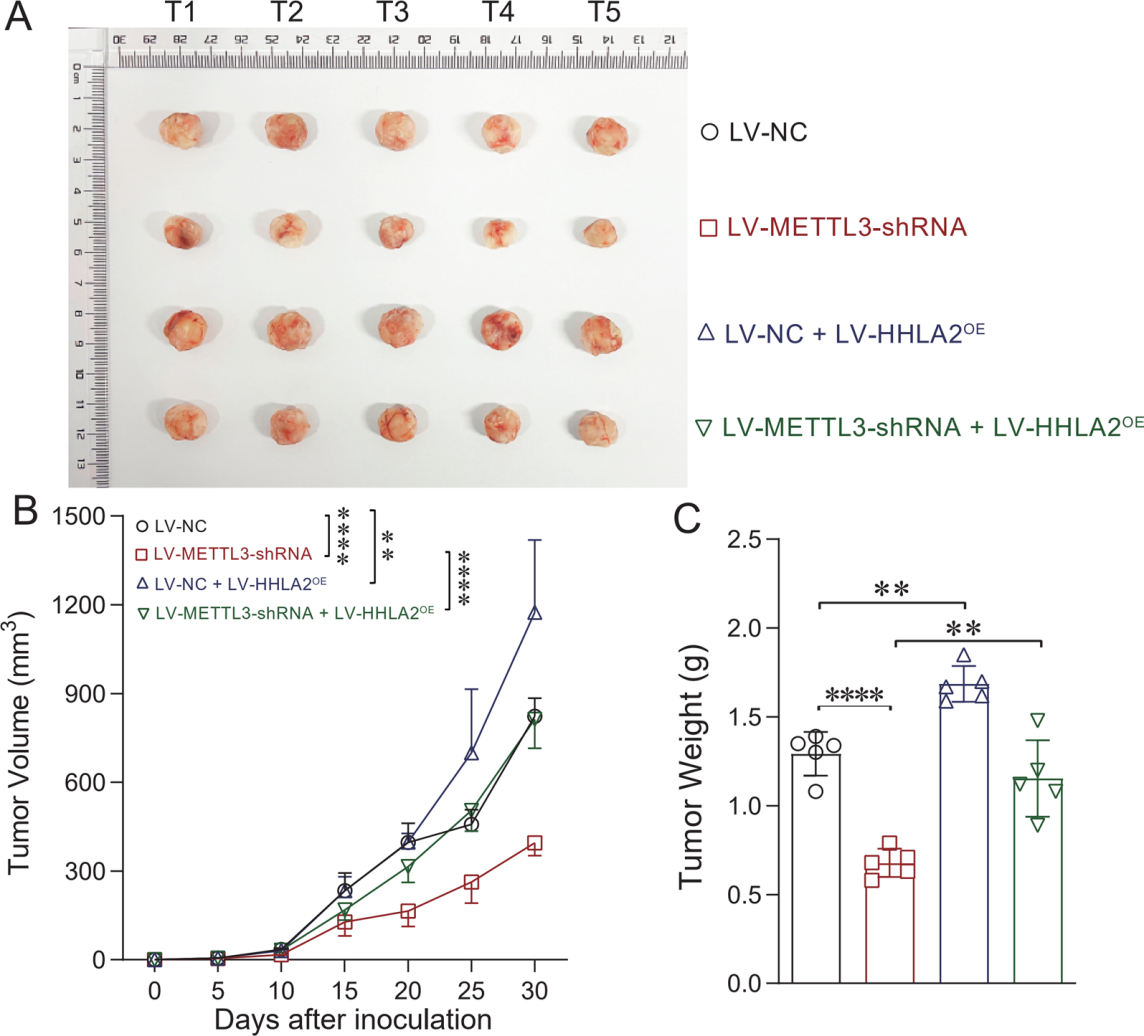
HHLA2 overexpression reverses the inhibition of tumor growth mediated by METTL3 depletion. The subcutaneous transplantation tumor model was used to study whether HHLA2 overexpression could reverse the inhibition of tumor growth mediated by METTL3 depletion. In 786-O cells, LV-NC, LV-METTL3-shRNA, LV-NC + LV-HHLA2^OE^, and LV-METTL3-shRNA + LV-HHLA2^OE^ subgroups (1 × 10^7^ in 150 µL PBS) were used to establish the tumor model in nude mice (4 ~ 5 weeks old). **A** and **B** Depletion of METTL3 could significantly decrease the tumor growth, and overexpression of HHLA2 could significantly increase the tumor growth. Overexpression of HHLA2 in the METTL3-depleted model could significantly reverse and promote the tumor growth. **C** The tumor weights of different subgroups were also evaluated. Consistent with the tumor growth curves, depletion of METTL3 could significantly decrease the tumor weights, while it could be reversed by overexpression of HHLA2 in 786-O cells. Un-paired *t* test and Two-way ANOVA analyses were used respectively. ^**^P < 0.01, ^****^P < 0.0001

The subcutaneous transplantation tumor model was used to study whether HHLA2 overexpression could reverse the inhibition of tumor growth induced by METTL3 depletion. LV-NC, LV-METTL3-shRNA, LV-NC + LV-HHLA2^OE^, and LV-METTL3-shRNA + LV-HHLA2^OE^ subgroups (1 × 10^7^ in 150 µL PBS) based on 786-O cell line were used to establish subcutaneous transplantation tumor model in nude mice (4 ~ 5 weeks old). Figure 6A and B show that depletion of METTL3 could significantly decrease the 786-O tumor growth (*P* < 0.0001), and overexpression of HHLA2 could significantly increase the 786-O tumor growth. Interestingly, overexpression of HHLA2 in the METTL3-depleted model could significantly reverse and promote the tumor growth of 786-O cells (*P* < 0.0001), suggesting that HHLA2 overexpression could reverse the inhibition of tumor growth induced by METTL3 depletion. The tumor weight measurement also confirmed this conclusion (Fig. 6C).

## Discussion

Increasing evidence suggests that abnormal expressions of m^6^A RNA methylation regulators are involved in the occurrence and development of human cancers, such as lung cancer, bladder cancer, nasopharyngeal cancer, and so on [22, 30, 31]. In human ccRCC, several reports have demonstrated that m^6^A regulators play important roles in cancer progression and prognostic prediction of the patients [3, 32, 33]. For example, Chen et al. have reported that METTL3 and METTL14, combined as a two-gene signature, can serve as an independent prognostic risk factor to distinguish ccRCC patients with different prognoses in both the training and validation sets, as well as their clinical datasets [34]. In our study, we found that METTL3 was highly expressed in human ccRCC tissues, and the ccRCC patients with low expression of METTL3 favored better OS compared with the patients with its high expression according to the IHC results. Moreover, based on our previous study [6], we also found that the expression level of METTL3 was significantly associated with HHLA2, suggesting that the underlying regulatory mechanism of METTL3 was dependent on the HHLA2 expression. The survival analysis also demonstrated that the OS of the patients with HHLA2^low^METTL3^low^ was significantly better compared with the patients with HHLA2^high^METTL3^high^, and the OS of the patients with HHLA2^low^METTL3^low^ also trended better than those with HHLA2^low^METTL3^high^, therefore these result further supported the notion that abnormal METTL3 expression in human ccRCC could not only serve as an important prognostic biomarker, but also could be suggested to contribute to the tumorigenesis of human ccRCC via potentially targeting HHLA2.

METTL3, as an important methyltransferase with a relative molecular weight of 70 kDa, has two critical domains to bind SAM and catalyze the formation of m^6^A [35, 36]. METTL3 can form a stable heterodimer core complex with METTL14, leading to cellular m^6^A deposition on nuclear RNAs [17]. Many reports have revealed that METTL3 contributes essentially to cancer progression and presents an important mechanism of epigenetic alteration in human carcinogenesis. For example, in human gastric cancer, up-regulation of METTL3 can promote tumor angiogenesis, glycolysis, and target MYC pathway, thus serving as a potential prognostic biomarker and therapeutic target for this malignancy [22]. In human hepatocellular carcinoma, METTL3 is frequently up-regulated and can regulate the expression of SOCS2 through an m^6^A-YTHDF2-dependent mechanism [37]. In human colorectal cancer, up-regulation of METTL3 can promote the metastasis of colorectal cancer cells via the miR-1246/SPRED2/MAPK pathway [38]. It has also been reported that METTL3 can promote translation of a large subset of oncogenic mRNAs, and METTL3 depletion can inhibit tumorigenesis and sensitize lung cancer cells to BRD4 inhibition ^[39]^. In the present report, our cellular study in human ccRCC cell lines also demonstrated that when METTL3 was depleted, the cell viability, migration ability, invasion ability, and tumor formation in vivo, were significantly inhibited. Therefore, all these data further confirmed that METTL3 might exist as a critical oncogene that promoted cancer progression.

Some studies have also revealed that m^6^A methylation regulators may be key mediators of PD-L1 expression and immune cell infiltration, which may strongly affect the tumor immune microenvironment ^[13, 40]^. Qiu et al. have reported that an important m^6^A demethylase, ALKBH5, can directly target PD-L1 mRNA, suggesting a novel regulatory mechanism of PD-L1 by mRNA epigenetic modification [11]. Based on our previous finding that HHLA2 was highly expressed in human ccRCC tissues and could serve as an important prognostic predictor [6], we also aimed to dissect the underlying mechanism of the regulation of HHLA2 expression in ccRCC cells. And in our present study, first, we found that METTL3 expression level was significantly and positively associated with HHLA2 expression, and the combination of METTL3 and HHLA2 expression in human ccRCC tissues could be used as an important predictor for poor prognosis, suggesting the potential role of METTL3 in the regulation of HHLA2. Second, it’s interesting that, based on our in vitro cellular investigation and in vivo tumor model study, we also demonstrated that overexpression of HHLA2 could significantly reverse the tumor inhibition mediated by METTL3 depletion. Third, our cellular results also showed that METTL3 could regulate the expression of HHLA2 via m^6^A modification of HHLA2 mRNA, leading to the mRNA stability, thus epigenetically increasing the expression of HHLA2 in ccRCC progression.

## Conclusions

In summary, our present study demonstrated that abnormal expression of METTL3 could serve as an important prognostic predictor for ccRCC patients, and we also indicated a novel regulatory mechanism of HHLA2 by mRNA epigenetic modification via METTL3. Moreover, we found that the METTL3/HHLA2 axis could promote tumorigenesis and progression of ccRCC.

## Supplementary Information

**Additional file 1:** **Figure S1.** Immunostaining of METTL3 and HHLA2 in adjacent normal renal tissues. **A.** Moderate METTL3 expression was found in adjacent normal renal tissue (scale bar=200 µm or 50 µm). **B**. Low expression of HHLA2 was found in adjacent normal renal tissue (scale bar=200 µm or 50 µm).

## Data Availability

All data generated or analyzed during this study are included in this published article.
