# Supplementary figures and images for: The methyltransferase METTL3 promotes tumorigenesis via mediating HHLA2 mRNA m6A modification in human renal cell carcinoma
Source: J Transl Med. 2022 Jul 6;20:298. doi: 10.1186/s12967-022-03496-3 (PMC9258210; doi:10.1186/s12967-022-03496-3)

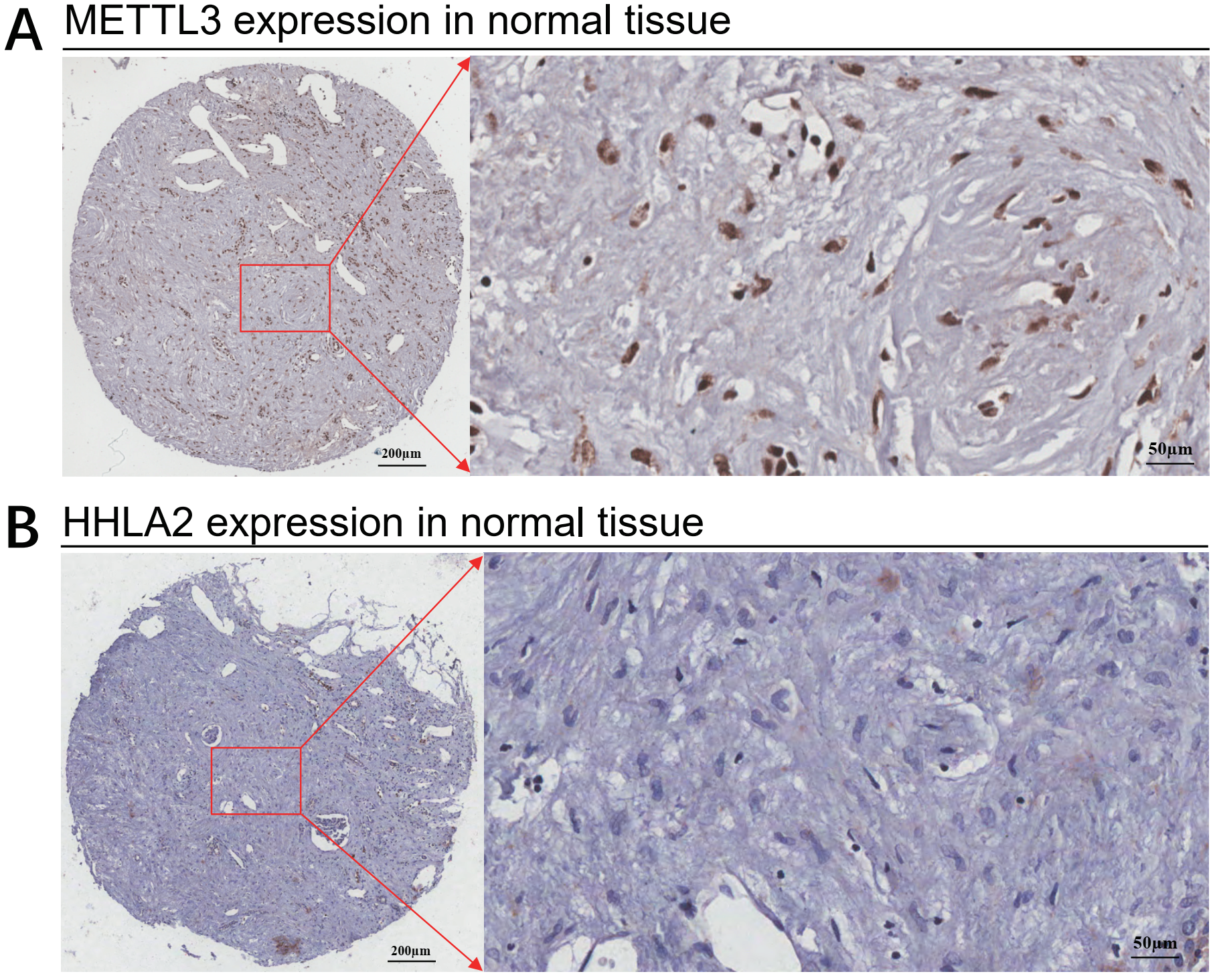

Supplement: Supplementary file 1 — Additional file 1: Figure S1. Immunostaining of METTL3 and HHLA2 in adjacent normal renal tissues. A. Moderate METTL3 expression was found in adjacent normal renal tissue (scale bar=200 µm or 50 µm). B. Low expression of HHLA2 was found in adjacent normal renal tissue (scale bar=200 µm or 50 µm). [file 12967_2022_3496_MOESM1_ESM.png]
